# Inhibition of human amylin aggregation by Flavonoid Chrysin: An *in-silico* and *in-vitro* approach

**DOI:** 10.7150/ijms.51382

**Published:** 2021-01-01

**Authors:** Abdullah A. Alkahtane, Hamzah A. Alghamdi, Bader Almutairi, Mohd Muazzam Khan, Md Saquib Hasnain, Mohamed M. Abdel-Daim, Wadha M. Alghamdi, Saad Alkahtani

**Affiliations:** 1 Department of Zoology, College of Science, King Saud University, Riyadh, Saudi Arabia.; 2 Department of Pharmacology, Faculty of Pharmacy, Integral University, Lucknow, India.; 3 Department of Pharmacy, Shri Venkateshwara University, NH-24, Rajabpur, Gajraula, Amroha - 244236, U.P., India.; 4 Pharmacology Department, Faculty of Veterinary Medicine, Suez Canal University, Ismailia 41522, Egypt.; 5 Medical Services at the Ministry of Interior, Riyadh, Saudi Arabia.

**Keywords:** Islet amyloid polypeptide, Chrysin, flavonoids, amylin aggregation

## Abstract

Islet amyloid polypeptide (amylin), consecrated by the pancreatic β-cells with insulin, has a significant role to play in maintaining homeostasis of islet cell hormones. Alzheimer's disease is the predominant source of dementia. However, its etiology remains uncertain; it appears that type 2 diabetes mellitus and other prediabetic states of insulin resistance contribute to the intermittent Alzheimer's disease presence. Amylin is abnormally elevated in Type II diabetes patients, accumulated into amylin aggregates, and ultimately causes apoptosis of the β-cells, and till date, its mechanism remains unclear. Several flavonoids have inhibitory effects on amylin amyloidosis, but its inhibition mechanisms are unknown. Screening a collection of traditional compounds revealed the flavone Chrysin, a potential lead compound. Chrysin inhibits amyloid aggregate formation according to Thioflavin T binding, turbidimetry assay. We report results of molecular interaction analysis of Chrysin with amylin which shows potent binding affinity against amylin. Pharmacokinetics and Drug likeness studies of Chrysin also suggest that it is a potential lead compound. Therefore, Chrysin prevented amylin aggregation.

## Introduction

According to a large number of studies, there is a strong association between genetic mutations, proteins dysfunction, environmental factors and neurodegenerative disorders 1. More evidence from epidemiological investigations, animal experiments, and *ex vivo* cell experiments showed that exposure to particulate matter (PM) may lead to neuro-inflammation, oxidative stress, mitochondrial dysfunction, neuronal apoptosis, synaptic damage and ultimately neurodegenerative diseases 2,3. Aberrant misfolding of proteins, as well as peptides into insoluble fibrils and aggregates, has indeed been related to multiple abnormalities such as Alzheimer's disease, Parkinson's disease, and type 2 diabetes mellitus (T2DM) 4. Amylin is a hormone of 37 amino acids long-chain, co-secreted with insulin from secretory pancreatic β-cell granules. Under T2DM-related hyperglycemic conditions, islet amyloid polypeptide (amylin) is susceptible to form oligomeric species and amyloidogenic aggregates that have been described to mediate dysfunction and apoptosis in pancreatic β-cells that secrete insulin and have been linked to insulin insufficiency in T2DM subjects 5-8.

Type 2 diabetes affects about 25.8 million Americans (about 8.3 percent of the population with almost 2 million new cases annually 9. Although the primary causes of type 2 diabetes remain uncertain, and the development of protein aggregates of the amyloid protein amylin is one of the factors leading to the progression of this disorder. These aggregates are potentially toxic to mammalian cells, in particular, the insulin-producing pancreatic β-cells 10. The prevention and the production of amylin is believed to slow, if not effectively stop, the progression of type 2 diabetes 11.

Alzheimer's disease is the leading neurological disorder in human beings, causing dementia, and so far, no drug or treatment has been found for the disease. Amylin aggregates are characteristic of several neurological disorders, which include diseases of such Alzheimer's, Huntington's and Parkinson's 4. Alzheimer's disease is a progressive form of dementia in postmortem brain tissues that is associated with neurofibrillary tangles and neurite plaques. These plaques contain extracellular aggregates of amyloid beta peptides whilst tangles are formed by intraneuronal accumulation of insoluble and hyperphosphorylated tau protein 12,13. Some research emphasized soluble amyloid-β oligomers as the primary toxic species in Alzheimer's disease 14. Different approaches were predicted to recognize inhibitors of amylin protein folding and aggregation, based on the above evidences, and to prevent or treat Alzheimer's disease. Examples include the use of small peptides (such as β-sheet blockers), 15-17, the use of agents that decrease amylin production 14,18, ways to increase amyloid-β clearance (amyloid vaccine) 19,20, and use of anti-oxidants 21,22.

The disease is exemplified by extracellular markers of amylin fibrils, hyperphosphorylated and misfolded tau protein intracellular neurofibrillary tangles, vascular injury inevitably results from substantial plaque accumulation and the deterioration of neuronal cells and synapses 23. Based on the most recent prominent hypothesis of neurodegenerative diseases, amyloid cascade, i.e., the deposition of amyloid-β peptide into plaques in brain tissue is the diseases causative agent 24. Produced from a single-pass transmembrane protein, amyloid precursor protein, amyloid-β is an amphiphilic and partially folded molecule, prone to self-aggregation, producing intermediate oligomers or protofibrils and ultimately insoluble fibrils 25. Amyloid-β impairs the functioning of certain membrane carriers, enhances cellular oxidative stress, and triggers neuroinflammation, resulting in substantial synapse dysfunction and loss of neurons 25,26.

It is known that at the molecular level, the naturally occurring plant ingredients exhibit a variety of functions as well as medicinally useful activities. Vegetables, spices, and herbal extracts have been reported as having beneficial health practices. The essential organic compounds in herbs, spices, and vegetables were studied for their antioxidant, anti-tumor, and anti-aging properties 9,27,28. Flavonoids are present in many plants, fruits, and vegetables and are considered as one of the most natural phytochemicals with a variety of pharmacological activities 29,30. These secondary metabolites were identified as potent antioxidants, free radical scavengers, and metal chelators 31-34 with anti-inflammatory 33,35, anticholinesterase 36 and neuroprotective properties 37,38. Besides, flavonoids can cross the blood-brain barrier with chronic or acute administration, indicating that these molecules could have a positive effect on the brain so that they can be used as prophylactics to delay the development of diseases like Alzheimer's as well as Parkinson's disease 39. Chrysin is a flavonoid that has drawn significant interest to its advantages in various conditions, which include neurodegenerative diseases, due to its numerous signaling pathways 40,41. Many reports highlighted the importance of flavonoids in neurodegenerative diseases 42-44. Therefore in the present study, we have reported the *in-vitro* activity of flavonoid Chrysin on the inhibitory potential of amyloid aggregation.

## Material and methods

### Chemicals/reagents

Chrysin (5,7-Dihydroxyflavone), Amylin, Congo red, Thioflavin T was purchased from Sigma-Aldrich, Inc. St Louis, MO, USA.

### Ligand Preparation

For computational simulation three dimensional (3D) structure of ligand molecule, Chrysin was retrieved from the PubChem database. Chrysin with PubChem ID (CID: 5281607) was used here for molecular interaction.

### Receptor Identification and Preparation

The human amylin structure was obtained from the Research collaborator for structural bioinformatics (RCSB) Protein data bank with PDB ID: 2L86 containing NMR structure. This PDB entry selected as it presents amylin in its native amidated form at physiological pH 45. Energy minimization of the fibril was done by the software Swiss PDB viewer 4.0.4.

### Molecular Docking Studies

A molecular docking study was performed to identify the binding pattern between flavonoid chrysin and hIAPP by using the software Autodock 4.2. Lamarckian genetic algorithm methodology was employed for docking simulations implemented in AutoDock 4.2 46. Here, 100 docking models are generated to search for the best interaction pattern of Chrysin towards the hIAPP. Using the AutoDock tools essential hydrogen atoms, Kollman united atom type charges, and solvation parameters were added. Affinity (grid) maps in *x, y* and *z* coordinates were set as 60×60×60 Å grid points and 0.375 Å spacing were generated using the autogrid program aimed to target grid co-ordinates inactive pocket of amylin fibril. Docking simulations were performed using the Lamarckian genetic algorithm (LGA) and the 'Solis & Wets local search method'. The population size was set to 150. The final figure was generated with the help of Discovery Studio Visualizer (Accelrys).

### Turbidity assay

Turbidity analyses were used to detect high-throughput screening false positives. To this, 50 μM amylin was mixed with 100 μM of the test compound. The kinetics of aggregation was monitored at an absorbance of 360 nm every 30 min using a Plate reader 47,48.

### Cell culture

C6, a rat glioma cell line, was procured from American Type Culture Collection (Manassas, VA, USA). The cells were cultured in tissue culture flasks and grown in Ham's F12 medium (Sigma Aldrich) supplemented with 10% Fetal bovine serum (FBS; Gibco, New Zealand), and 1% antibiotic antimycotic solution (Sigma-Aldrich) in a humidified atmosphere of air and 5% CO2 at 37 °C.

### Cytotoxicity assays

The in-vitro cytotoxicity of naringin was determined following the protocol by 3-(4,5-Dimethylthiazol-2-yl)-2,5-Diphenyltetrazolium bromide (MTT) (Sigma-Aldrich) assay 49. Briefly, in 96 well plates, C6 cells were seeded and incubated for 24 hours. Semi-confluent cells have been exposed to increasing chrysin concentrations for 24h. Cells were added per well with 10 μl of MTT solution for MTT analysis and incubated at 37 °C for 4h. 100 μl of solubilization solution was applied to each well after incubation and well mixed to dissolve formazan crystals. Absorption was taken with a microplate reader at 570 nm. The IC50 of Chrysin was found to be 71.237 µM.

### Thioflavin T fluorescence measurement

C6 cells were maintained in DMEM supplemented with 5 mM D-glucose, 10% FBS, and 1% antibiotic/antimycotic solution in a humidified atmosphere of air and 5% CO2 at 37 °C. C6 cells were subjected to the treatment of amylin (25 nM) with or without chrysin (50 µM) for 24 hours. Cells were washed with PBS and added Thioflavin T (ThT) dye (5 µM). Fluorescence was determined at excitation and emission wavelengths 440 nm and 482 nm respectively.

### Congo red staining

C6 cells were subjected to the treatment of amylin (25 nM) with or without chrysin (50 µM) for 24 hours. Cells were washed with PBS and added congo red dye. After incubation for half an hour, the image was taken under a fluorescent microscope EvosFLc Microscope (Thermo-Scientific).

### Data Analysis

Results were expressed as Mean ± SEM. Multiple data comparisons involving more than two groups and/or time points were analyzed by one-way ANOVA followed by Tukey's multiple comparisons tests using GraphPad Prism 7.0 software (GraphPad Software, San Diego, CA, USA). Data comparison involving two groups was analyzed using the two-tailed unpaired Student *t*-test. *p*<0.05 value was set as statistically significant.

## Results

### Molecular Docking

**Figure 1:**
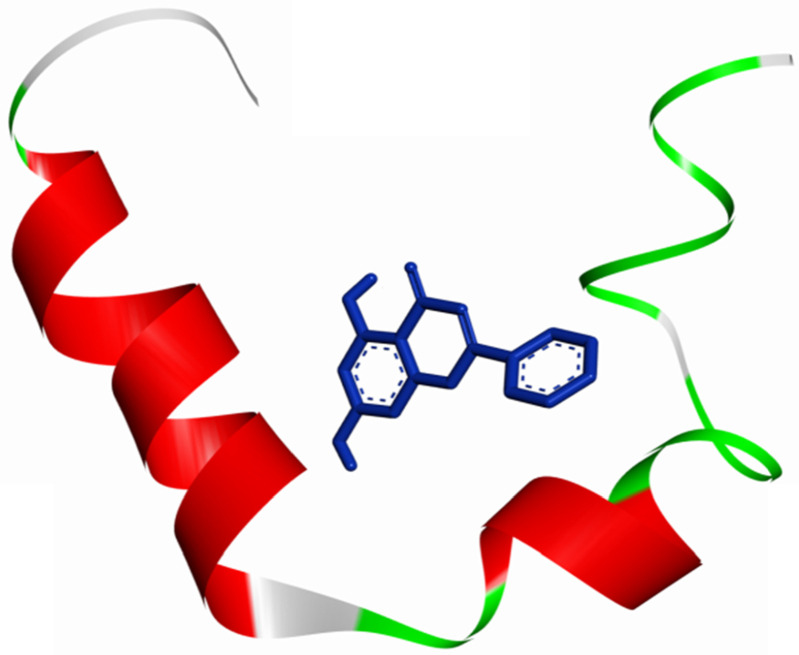
An overall view of the molecular docking pose of Chrysin into the binding site of hIAPP. Chrysin interact in the active pocket of amylin (human islet amyloid polypeptide).

**Figure 2:**
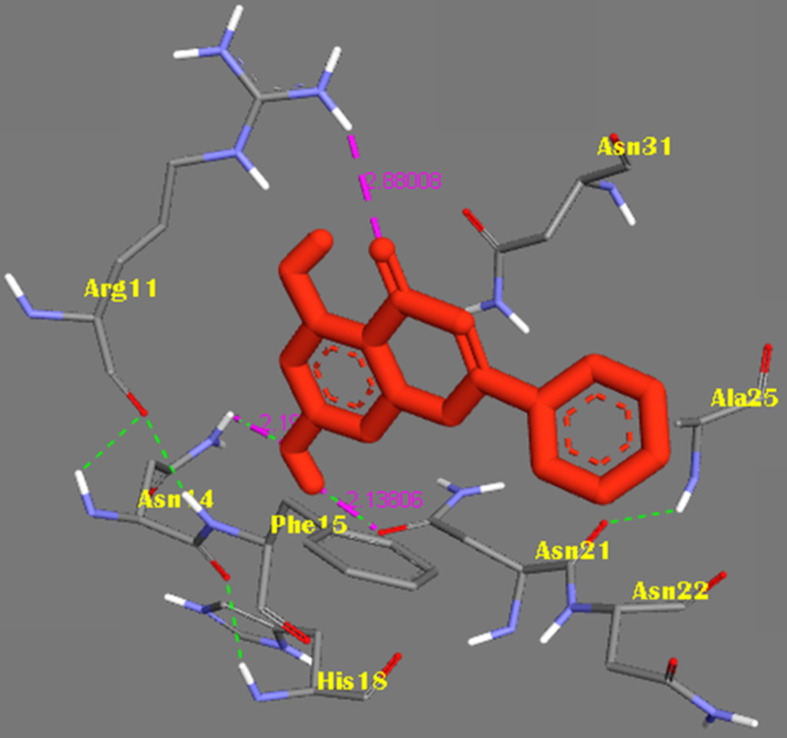
Representation of the binding mode between Chrysin and hIAPP. Chrysin molecule was represented by RED color; hIAPP was represented in BLUE-GREY stick representation surrounded the Chrysin by its active interacting amino acid residues in YELLOW. Hydrogen bonds were shown as GREEN dots. Intermolecular H-bonds were shown with their respective bond lengths in PINK.

The molecular docking method was proposed for the visual identification of the binding pose of amylin with its inhibitor. Chrysin was found to interact in the active pocket of amylin the human islet amyloid polypeptide as shown clearly in figure 1. Maximum binding affinity between amylin and chrysin was found to be -6.45 Kcal/mol with 23.25µM Ki (inhibition constant) shown a strong interaction between the two as data shown in **Table 1.** Residues Arg11 and Asn14 were found to anchor the chrysin molecule at the active binding site of amylin. While it is predicted that residues Phe15, His18, Asn21, Asn22, Ala25, and Asn31 might help in the efficient interaction. Chrysin forms three strong H-bonds with the amylin respectively at the positions O3 of chrysin with Arg11 (position) of amylin with a bond length of 2.88 Å, at O4 of chrysin with Asn14 of amylin with a bond length of 2.19 Å and at OD1 of chrysin with UNK0 position of amylin with a bond length of 2.13 as shown in **Figure 2.**

### Effect of Chrysin on amylin aggregation

**Figure 3:**
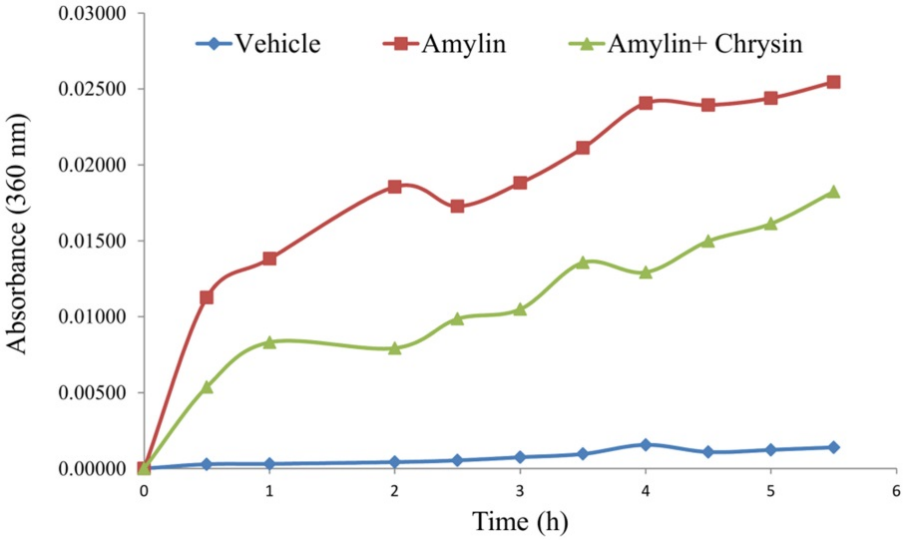
Effect of Chrysin on amylin aggregation measured at 360 nm for 5.5h. The amylin aggregation was determined by the turbidity assay technique. The amylin forms aggregate with an increase in time, as shown by the kinetic study performed. The test molecule Chrysin inhibited the aggregation fibril formation.

*In vitro,* the amylin aggregation was determined by the turbidity assay technique. The amylin forms aggregate with an increase in time, as shown by the kinetic study performed. The test molecule Chrysin inhibited the aggregation fibril formation (**Figure 3**).

### Effect of Chrysin on amylin aggregation by ThT assay and congo red staining

**Figure 4:**
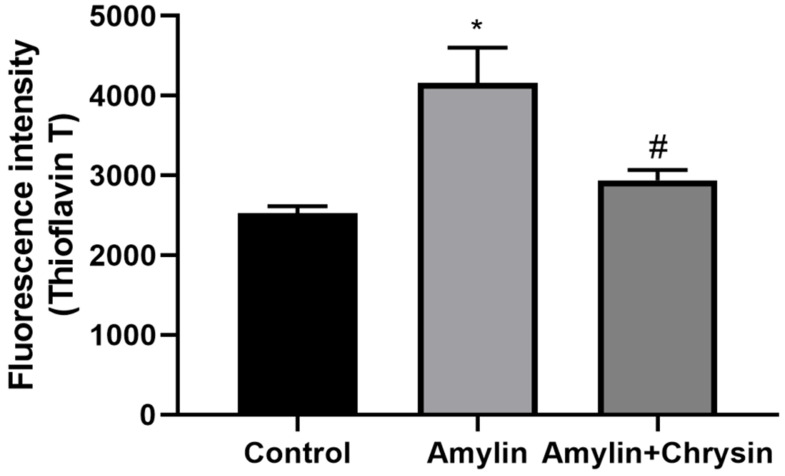
Effect of Chrysin on amylin aggregation by using Thioflavin T (ThT). Cells exposed to amylin shows increased fluorescent intensity. This increase in amylin aggregation was decreased by the treatment of Chrysin. The fluorescence intensity was measured at excitation and emission wavelengths 440 nm and 482 nm.

**Figure 5:**
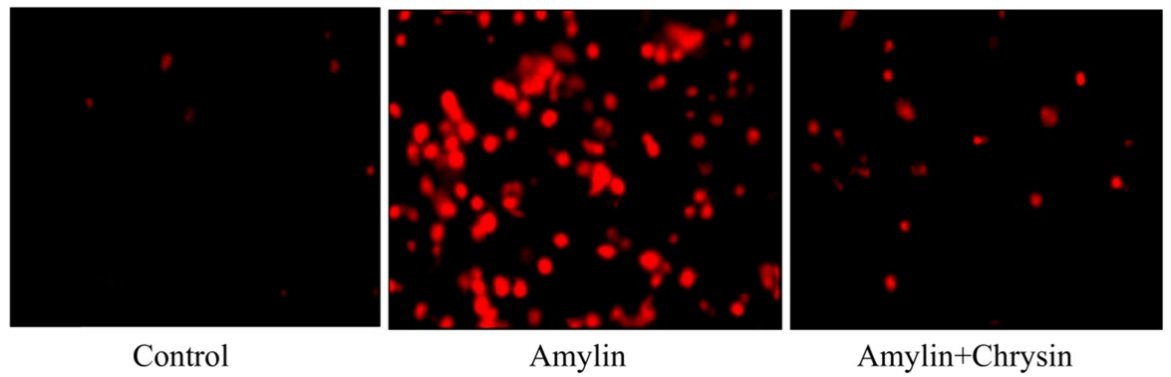
Effect of Chrysin on amylin aggregation by using Congo red dye. Cells exposed to amylin shows enhanced amylin aggregation. This increase in amylin aggregation was decreased by the treatment of Chrysin.

The ThT assay, as well as congo red staining, was performed in C6 cell lines. Cells exposed to amylin shows increased fluorescent intensity using dye ThT and enhanced amylin aggregation using Congo dye. This increase in amylin aggregation was decreased by the treatment of Chrysin, as depicted in **Figures 4** and **5.**

## Discussion

Since amylin is considered as the initiator of events leading to neurotoxicity and the clinical symptoms of Alzheimer's disease, the hunt for anti-amyloid therapies has become a significant strategy in Alzheimer's disease-related study 50-52. The potential therapeutic approaches have been so far proposed to reduce the production of the amyloidogenic form of proteins, to increase the clearance rate of misfolded or aggregated proteins or to stabilize the native state of amyloidogenic proteins, as well as to directly inhibit the self-assembly process. At the moment, the focus is put on regulating the prefibrillar conformers (soluble oligomers) of amylin proteins, as these are typically the most toxic aggregates *in vitro* and *in vivo* 4,53-55. Rather than targeting all possible intermolecular contacts, the use of small molecules such as sugars, polyols, amino acids, amines, salts, polymers and surfactants that interrupt specific intermolecular contacts could be a better way to alter the nucleation pathway and prevent the formation of fibrils. Flavonoid, strongly linked to quercetin, has already been identified as inhibiting the aggregation of amylin as well as disaggregating its fibres 55,57. Quercetin prevents amylin aggregation and partially safeguards RIN-m5F cells from damage caused by extracellularly incorporated amylin 48. There is growing interest in herbal medicine today, as they are also viewed favorably as more natural therapies than prescription drugs. In particular, successful dementia treatments have been developed from herbal medicines 58.

Studies suggest that both anti-oxidative and anti-apoptotic properties of chrysin (especially in the dose of 100 mg/kg) are possible mechanisms that improve cognitive/motor deficits and prevent neuronal cell death after traumatic brain injury 59. This peptide has played a central role in Alzheimer's studies ever since exploration that perhaps the amylin peptide is the chief component of the fibrils present in the extraneuronal senile neuritic plaques in the brains of Alzheimer's patients. The soluble oligomers are now considered to be neurotoxic, and the mature fibrils of the amyloid beta-peptide are not per se neurotoxic. Some believe that the mature amylin fibrils may serve as a reservoir of amyloid beta-peptide soluble oligomers. Thus, there seems to be scientific proof that, following oligomerization, huge aggregates may release smaller aggregates reversibly. After high-speed centrifugation of brain extracts, soluble amylin peptide oligomers are identified as what remains in aqueous solution.

Amyloidosis is correlated with the largest class of protein misfolding diseases which involves a wide range of neurological, metabolic, and aging-associated conditions including Alzheimer's disease, prion disease, Parkinson's disease, and type 2 diabetes diseases. The distinguishing pathological features of amylin are intracellular and extracellular insoluble protein deposits called amyloid fibrils 4,60,61. The amylin fibrils are formed with time (**Figure 3**) and appear to be turbid. The aggregation of amylin proteins progresses through a nucleation-dependent cycle in which monomeric and oligomeric aggregates form “seeds” that induce a cascade of aggregation resulting in a balance between mature amyloid fibrils and their small precursor aggregates. Mature amyloid fibrils consist of several unbranched segments of the protofilament, which in turn are composed of β-sheet rich protein structures. Both structures stack each other, creating the conserved “cross beta spine” amyloid, distinguished by individual β-strand units perpendicular to the protofilament's long axis 4. This formed amylin fibril or turbidity is inhibited by the addition of Chrysin, which shows that the flavonoid Chrysin inhibits the amylin fibril formation due to its antioxidant activity (**Figure 3**).

The formation of neurotoxic oligomers and protofibrils are the main steps leading to plaque formation in the central nervous system, which results in Alzheimer's disease neurodegeneration. Insoluble amylin oligomers and protofibrils have been shown to have a cytotoxic effect compared to soluble peptides when incubated with *in vitro* neuronal cells 62. Besides, it was also shown previously that amylin induces the production of reactive oxygen species 63. Based on these findings, amylin peptides are suggested as appropriate therapeutic targets in disease-like Alzheimer's disease that may have a disease-modifying effect. The amylin aggregation takes place as revealed by the Thioflavin T assay, the fluorescence intensity of the amylin treated group shows increased fluorescence intensity as compared to the control group (*p* < 0.05) (**Figure 4**). Simultaneously the congo red stained cells depicts enhanced intensity as compared to the control group, which reveals that amylin form aggregates in C6 glioma cells when treated with amylin (**Figure 5**). Thus on treatment with Chrysin, it inhibits the aggregate fibril formation and gives less fluorescence intensity with Thioflavin T (*p* < 0.05) (**Figure 4**) and produces a low intensity of fibril aggregates when treated with Chrysin in congo red binding assay (**Figure 5**). In regards to protein concentration, numerous factors may contribute to amyloidogenic aggregation, such as cellular environment and genetic mutations. For the pathologic amylin amyloid formation implicated in T2DM, no inhibitors have yet been clinically approved, making the prevention of amylin amyloid formation a very active area of research 64. As with the potent inhibitors, chrysin, the amylin lag step extension, when incubated with inhibitor, indicates that inhibitors are likely to interfere with amylin protein at an early stage in the aggregation process to minimize amyloidogenic fibril formation. Chrysin can act on destabilized monomeric amylin and slow down the transition to larger oligomeric structures, which, in turn, are precursors to amylin amyloid fibrils. Chrysin reduces the risk of developing T2DM. The administration at an early stage by their antioxidant properties, which would enable them to scavenge the reactive oxygen species (ROS) induced by toxic amylin and inhibit the deposition of amylin aggregates in pancreatic islets and prevent their loss. T2DM is a chronic disease in which disease progression, amylin accumulation, and deposition 65,66 require a prolonged time. Regular ingestion of chrysin, through diet or supplements, may protect against toxic amylin aggregation and the consequent death of β-cells.

The future therapeutic strategy involves avoidance of insoluble development of amylin, inhibition of oligomerization, and formation of fibrils, clearance of insoluble amylin peptides and, therefore, neurotoxicity prevention. Several scientists have shown the anti-amyloidogenic ability of several Phyto-compounds in light of the amylin hypothesis. One compound namely multimeric derivatives of quinacrine inhibit the formation of amylin fibrils 67. Further research showed that carbazole-derivatives prevented the development of amylin fibrils *in vivo* 68. It has been suggested that the compounds that bind to amylin peptides could be useful against formations of amylin fibrils.

## Conclusion

Thus, by inhibiting amylin fibril aggregate formation under *in vitro* environment, chrysin demonstrated considerable anti-amylin potential, and this study gives a substantial lead that chrysin has the potential to be used as an anti-aggregation agent that could have disease-modifying effects in Alzheimer's disease. In addition, animal studies and pharmacokinetic studies are needed to validate *in vivo* models of the anti-amylin fibril aggregate potential.

## Figures and Tables

**Table 1:** Binding affinity between amylin and chrysin

Target	Compound Name	Binding Energy (ΔG)	Inhibition Constant Ki	Interacting Amino Acids	No. of H-bonds	No. of intermolecular H-bonds
Human islet amyloid polypetide (amylin)	Chrysin	-6.45 Kcal/mol	23.25µM	Arg11, Asn14, Phe15, His18, Asn21, Asn22, Ala25, Asn31	9	3

## Abbreviations

T2DM: Type-2 diabetes mellitus
RCSB: Research collaborator for structural bioinformatics
LGA: Lamarckian genetic algorithm
FBS: Fetal bovine serum
MTT: Dimethylthiazol-2-yl)-2,5-Diphenyltetrazolium bromide
ThT: Thioflavin T
ER: endoplasmic reticulum
PCA: protocatechuic acid
